# Alternative intraoral donor sites to the chin and mandibular body-ramus

**DOI:** 10.4317/jced.54372

**Published:** 2017-12-01

**Authors:** David Reininger, Carlos Cobo-Vázquez, Benjamin Rosenberg, Juan López-Quiles

**Affiliations:** 1 DDS, Master in Oral Surgery and Implantology. Instructor Professor, Departament of Oral and Maxillofacial Surgery, Universidad de los Andes; 2 PhD, DDS, Master in Oral Surgery and Implantology, Universidad Complutense de Madrid; 3 DDS; 4 DDS, MD, PhD, Maxillofacial Surgeon, Associate Professor, Department of Oral Surgery and Maxillofacial Surgery, Universidad Complutense de Madrid

## Abstract

**Background:**

Provide a review of alternative intraoral donor sites to the chin and body-ramus of the mandible that bring fewer complications and that may be used to regenerate small and medium defects.

**Material and Methods:**

A review was conducted using the search engine PUBMED and looking manually into scientific journals.

**Results:**

From the 35 articles included, 6 corresponded to the coronoids, 3 corresponded to the zygomatic body, 5 corresponded to the anterior maxillary sinus wall, 3 corresponded to the zygomatic alveolar process, 2 corresponded to the incisive fossa, 2 corresponded to the anterior nasal spine, 2 corresponded to the palatal region, 5 corresponded to the tuberosity, and 7 corresponded to the palatal and mandibular tori.

**Conclusions:**

Although there are few complications described when using alternative intraoral donor sites, the main problem with these types of grafts is their scarce bone volume, with only the zygomatic body, anterior sinus wall, and palate sites being able to be used in medium defects. More clinical trials are necessary in order to evaluate the behavior of the alternative donor sites over time.

** Key words:**Grafting, autologous bone, autografts, mandible, maxilla, palate hard, zygoma.

## Introduction

There are several factors that cause the resorption of the alveolar process or in more severe cases the resorption of the basal bone. They include bone loss as a consequence of trauma, due to tumor surgery, due to periodontal pathology, or as a result of the resorption following dental extraction itself. In the latter case, specifically, according to a systematic review from 2012 (1), a horizontal resorption of 3.79 +/- 0.23mm and a vertical resorption of 0.24 +/- 0.11mm were described, the loss of bundle bone being the factor triggering resorption (2). This resorption occurs more severely during the first 6 months, there is higher resorption in the vestibular cortical plate, and a residual bone resorption range of 0.1mm per year for the maxillary area and of 0.4 mm per year for the mandibular area (2) is estimated. Furthermore, the degree of bone resorption can be increased due to anatomic, metabolic, functional, and prosthetic factors (3,4).

Depending on severity, the resorption could cause functional and aesthetic alterations, with incorrect crown-to-implant ratios due to excessively long crowns compromising the aesthetic aspect of rehabilitation, or in cases of more severe resorption the implant installation could be prevented. Most of these complications can be dealt with using regenerative techniques, techniques that are applied when installing the implant or before, with autologous bone, allografts, xenografts, or alloplastic grafts, in block or particulate form, with or without membrane, which can be resorbable or non-resorbable (5,6). Among all bone alternatives, autologous bone continues to be the gold standard in bone regeneration (7,8), due to its osteogenic, osteoinductive, and osteoconductive properties, in addition to its growth factors and the fact that it does not cause immunogenic reactions (7).

Autologous grafts are classified according to their origin being intraoral or extraoral, and according to their embryologic origin being endochondral or membranous (7). In general, extraoral grafts are used in large defects, while intraoral grafts are used in medium or small defects. The main advantages of intraoral grafts are the following: they are located near the recipient site, they reduce operative time, they allow using a lower amount of anesthetic, they involve less morbidity and discomfort in the patient, and they allow the use of local anesthesia and do not require hospitalization (7,9,10). The most frequently used intraoral donor sites are the chin and body or ramus of the mandible, the main disadvantages of which are their high number of postoperative complications (11-13), including sensitivity alterations in the teeth, mucosa or skin, alterations that can be temporary or permanent; opening limitations; and facial outline alterations.

Therefore, the objective of this study is to review, analyze, and compare the different intraoral donor sites that are used in the maxillofacial region, specifically at alveolar level, and that may provide alternative donor sites with a lower rate of postoperative complications.

## Material and Methods

A review of literature from 1990 to march 2017 was conducted using the PUBMED database and looking manually into the following journals: Journal of Oral and Maxillofacial Surgery, International Journal of Oral and Maxillofacial Surgery, Clinical Oral Implants Research, Clinical Implant Dentistry and Related Research, Journal of Oral Implantology, and International Journal of Oral and Maxillofacial Implants. The inclusion criteria for the studies were: studies conducted between 1990 and 2016, both prospective and retrospective, in English or Spanish, conducted on humans, indicating clearly the site from which the graft comes and the technique for extracting the graft and commenting on any complications that may have occurred. All the cadaver studies or studies on patients with uncontrolled metabolic diseases, having undergone radiotherapy to the head area during the last 24 months or treatment with bisphosphonates given intravenously or orally during 3 years or more, with psychiatric problems, and/or with a heavy smoker (more than 10 cigarettes a day), heavy drinker or drug user profile were automatically excluded.

The search was conducted using the following MESH terms: “Bone Transplantation,” “Transplantation, Autologous,” “Auto-grafts,” “Mandible,” “Maxilla,” “Palate, Hard,” “Zygoma,” in the following form: (“Bone Transplantation”[Mesh] OR “Transplantation, Autologous”[Mesh] OR “Autografts”[Mesh] AND (“Mandible”[Mesh] OR “Maxilla”[Mesh] OR “Palate, Hard”[Mesh] OR “Zygoma”[Mesh]), then adding the filters between 1990 and September 2015, human beings, English or Spanish language.

Two reviewers (D.R. and C.C.) examined independently the titles and abstracts of all the references selected in the initial search. All studies to be selected had to meet the inclusion criteria; in those cases in which reading the title and the abstract was not enough to know if said criteria were met the whole texts were read. Thus, a complete list of the articles to be fully read by each reviewer separately was made. Then, the reviewers read the full articles and selected the studies that were going to be included in the final review. In cases of disagreement on a specific article, a third person (J.L.Q.) joined the original two reviewers in a discussion to decide. In order to avoid selection bias the authors hid the name of the journal, the institutions and names of the authors for the review.

## Results

**Figure 1:**
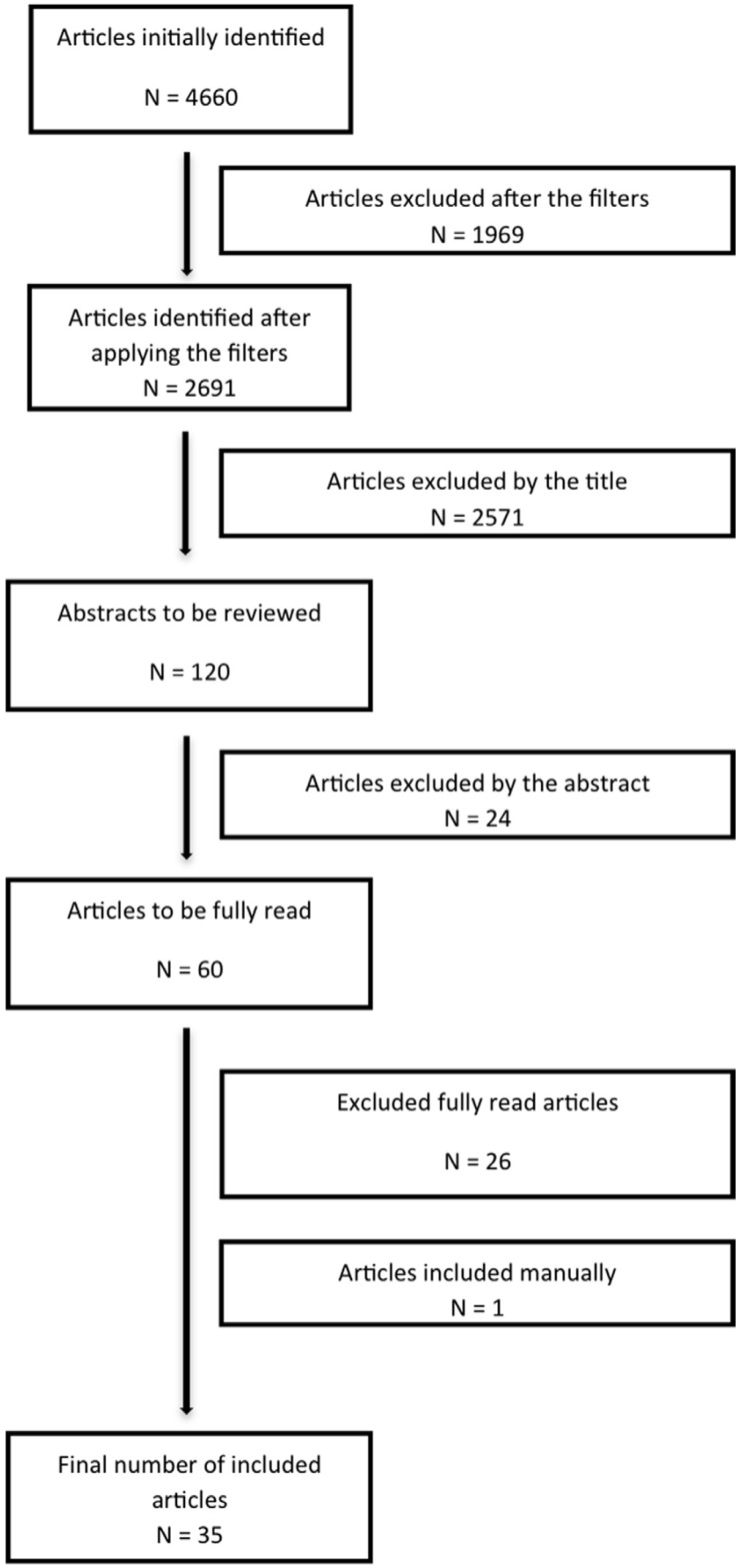
Flux diagram of the search and selection process.

The initial search resulted in a total number of 4,660 articles, which then were reduced to 2,691 after the application of the filters. These 2,691 articles were reduced to 120 with only the abstracts, with a coincidence percentage of 94.6% between the reviewers. A total of 60 articles were fully read, of which 34 were finally selected, with a coincidence of 100% between the reviewers. The search was complemented with the manual review, which resulted in the addition of one article from the Journal of Oral and Maxillofacial Surgery (Fig. 1).

When analyzing the results of each donor site independently ( Table 1,  Table 1 continue):

**Table 1:**
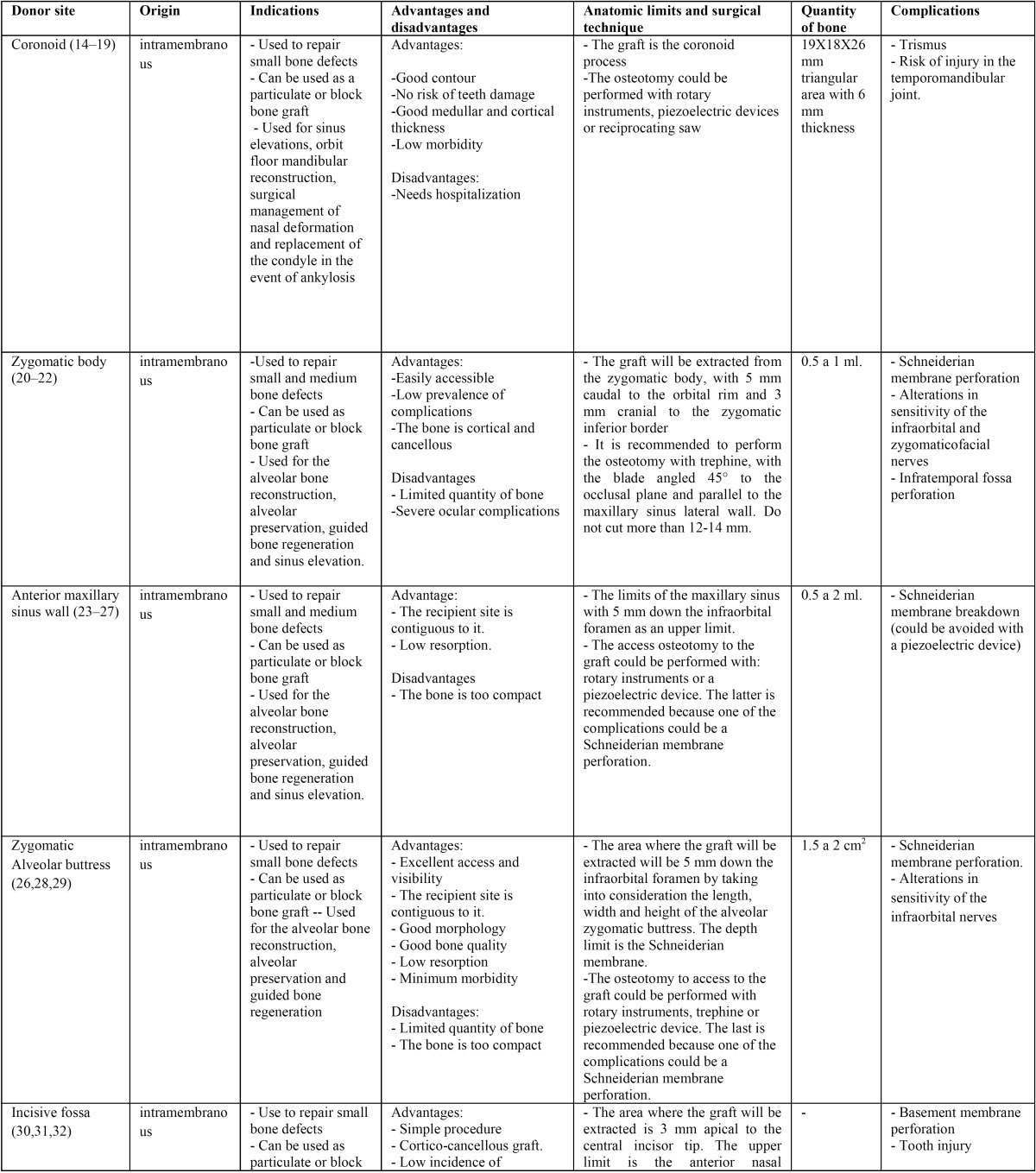
Description of the different intraoral donor sites.

**Table 1 continue:**
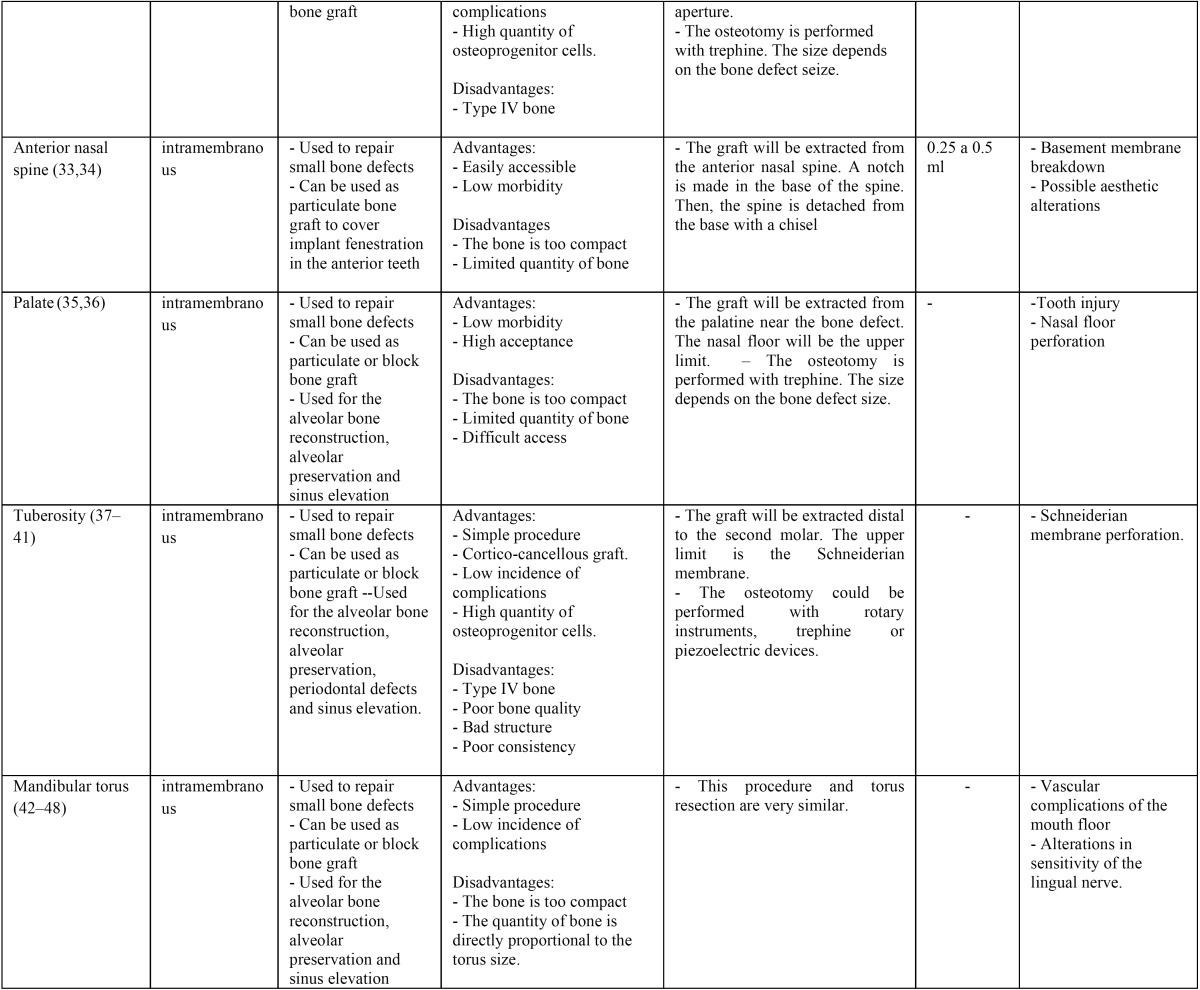
Description of the different intraoral donor sites.

I.- Coronoid process:

Of the total amount of articles, 6 correspond to the use of the coronoids process as donor site (14-19), with which a total of 131 patients were treated. The graft was applied in block form in 99.3% of the cases and in particulate form in the remaining 0.7%. The goal of the surgery was auricular reconstruction in 49.6% of the patients, paranasal increase in 41.2%, orbital reconstruction in 3.8%, mandibular reconstruction in 1.5%, alveolar ridge increase in 1.5%, anterior sinus wall reconstruction in 0.7%, chin increase in 0.7%, and maxillary sinus elevation in 0.7%. All the surgeries were performed under general anesthesia, and, for the osteotomy, cylindrical burrs were used in 10% of the cases and reciprocating saws in the remaining 90%.

II.- Zygomatic body

3 articles (20–22) were included, in which 36 patients were treated. The graft was applied in block form in 2.7% of the cases and in particulate form in the remaining 86.1%. It was used for the regeneration of fenestrations at the moment of installation of the implant in 66.6% of the cases, for sinus elevation in 30.5%, and for closing oral-sinus passage in 2.7% (in the graft it was used in block form). Surgeries were performed under nitrate oxide sedation in 61.1% of the cases, under local anesthesia only in 25%, and under general anesthesia in the remaining 2.7%. The osteotomy was performed with rotatory instruments in 66.6%. of the cases, with implant burrs in 22.2%, and with trephine in the remaining 11.1%.

III.- Anterior maxillary sinus wall

5 articles (23–27) were selected, in which a total of 138 patients were treated. The graft was applied in particulate form in 73.2% of the cases and in block form in the remaining 26.8%. It was used for sinus elevation in 64.8% of the cases, for orbital floor reconstruction in 19.3%, for implant deshiscences in 8.2%, and for horizontal increases in the remaining 7.5%. The graft was extracted with bone scrapers in 73.1% of the surgeries, with burrs in 19.3%, and with piezoelectric scalpel in the remaining 7.5%. 73.2% of the surgeries were performed under local anesthesia only and 26.8% of them under general anesthesia.

IV.- Zygomatic alveolar process

3 articles (26,28,29) were included, in which a total of 66 patients were treated. The graft was applied in block form in 97.1% of the cases and in particulate form in the remaining 2.9%. It was used for sinus elevations in 77.6% of the cases, for the regeneration of deshiscences posterior to the installation of the implants, and for horizontal regeneration in the remaining 2.9% of the cases. The graft was extracted with bone scrapers in 97.1% of the surgeries and with piezoelectric scalpel in the remainint 2.9%. All patients were treated with local anesthesia.

V.- Incisive fossa

2 articles (30,31,32) were included, in which 2 patients were treated. The grants were used for the regeneration of a bone fenestration and for alveolar preservation. All procedures were performed with local anesthesia and the graft was used in particulate form.

VI.- Anterior nasal spine

2 articles (33,34) were included, in which 16 patients were treated. The grafts were used for the regeneration of fenestrations and deshiscences at the moment of installation of the implant. 93.7% of the patients were treated with intravenous sedation and 6.2% of them were treated only with local anesthesia. In all cases the base osteotomy was performed with rotatory instruments.

VII.- Palatal region

2 articles (35,36) were selected, in which a total of 19 patients were treated. The graft was applied in block form in 94.6% of the cases and in particulate form in 5.4%. It was used for alveolar preservation in 89.4% of the cases, for horizontal regeneration in 5.2%, and for sinus elevation in 5.2%. All procedures were performed with trephine and under local anesthesia.

VIII.- Tuberosity

5 articles (37–41) were selected, in which a total of 44 patients were treated. The graft was applied in particulate form in 52.3% of the cases and in block form in the remaining 47.3%. It was used for horizontal regeneration in 61.3% of the cases, for the regeneration of fenestrations or deshiscences in 36.5%, and for sinus elevation in the remaining 2.2%. The osteotomy was performed with rotatory instruments in 61.3% of the cases, with trephine in 36.5%, and with a saw in the remaining 2.2%. 97.8% of the patients were treated only with local anesthesia, while 2.2% of them were treated with general anesthesia.

IX.- Torus

A total of 7 articles (42–48) were selected, where 9 patients were treated, the tori being from the palatal area in 88.8% of the cases and from the mandibular area in the remaining 11.2%. The graft was applied in block form in 50% of the cases and in particulate form in the other 50%. It was used for horizontal increases in 80% of the surgeries, for periodontal regeneration in 10%, and for sinus elevation in the other 10%. Rotatory instruments were used in 100% of the cases, of which 80% were treated only with local anesthesia, 10% with local anesthesia and sedation, and the remaining 10% with general anesthesia.

## Discussion

A small defect will be defined as that whose maximum dimensions are 7mm of length, 5mm of depth, and 12mm of height, dimensions corresponding to an alveolus. A medium defect will be that corresponding to a length of 2 to 3 teeth, and whose dimensions correspond to 14–21mm of length, 5mm of depth, and 12mm of height. A large defect is that which surpasses 3 teeth, with a length of over 21mm. Regenerating a small defect requires a volume of 0.42ml, medium defects require between 0.84 and 126ml, and in large defects require volumes greater than 1.26ml approximately (20).

Intraoral grafts are indicated to regenerate small and medium defects, the chin and body-ramus being the most used donor sites. In a cadaver study Yates *et al.* (42) mention that the chin region corresponds to an average volume of 1.15ml and the mandibular body-ramus corresponds to one of 2.02ml. A study conducted by Verdugo *et al.* (43) evaluated radiographically and clinically the volume obtained when using the mandibular body-ramus, obtaining an average of 0.8ml radiographically and of 2.5ml clinically. Later on, Verdugo *et al.* (44) conducted the same study, but using the chin region, obtaining an radiographic average volume of 1.4ml and a clinical one of 2.3ml. In both cases the difference between both measurements was attributed to the size of the particle on which the measurement was performed. In the specific case of the body-ramus the difference is also determined because the measurement was performed only on cortical tissue, whereas clinically both cortical and spinal tissue was obtained. Although both donor sites correspond to volumes suitable for the reconstruction of small and medium defects, their main disadvantage lies on the several postoperative complications they cause (11–13), which led to the search of new intraoral donor sites that could regenerate these types of defects.

When evaluating each donor site independently:

1.- Coronoid process: it is a site used mostly for orbital floor reconstruction, paranasal augmentations and TMJ reconstruction. Due to the need to operate under general anesthesia, its complicated access, and the presence of alternative donor sites providing higher bone quantity and better access, this site is of scarce utility in maxillary reconstruction for implantologicpurposes (14–19).

2.- Zygomatic body: donor site used mainly in particulate form. According to the articles included it is usually used in implants. One of its advantages is that it is located near the antral teeth. It could be very useful to close oroantral communications as in the technique described by Peñarrocha *et al.* (22) or Nurray *et al.* (45). Kainulainen *et al.* (20) determined in a cadaver study an average volume of 0.53 ml, with 37.5% sinus membrane perforation and 17.5% infratemporal fossa perforation. Another complication described is the presence of a hematoma (20–22). Keinulainen *et al.* (21) point out that by comparing this donor site and the chin, the surgery is more comfortable and less morbid for the patient. According to the volumes obtained in these studies, this type of graft is used for small and medium defects.

3.- Anterior maxillary sinus wall: wide donor site but narrow, mainly cortical. It is ideal for use in sinus elevations due to its location. While completely separating the membrane from the bone window, the risk of perforation is high; this can be diminished using a piezoelectric scalpel (46). Also, small perforations present a good prognosis, without compromising the final result of the elevation (46). Besides, it is a bone with an ideal thickness to perform the box technique (25). Its anatomy is ideal for orbit floor reconstructions (23,27).

4.- Alveolar zygomatic buttress: easily accessible area, used mainly for small defects. It is an ideal donor site due to its location. In spite of always being used in particulate form in the aforementioned studies (mainly because of the bone thickness), we propose to use this graft as a block in small defects extracting the graft with a piezoelectric scalpel s or trephine, always after a detailed radiologic study. As with the aforementioned case, the main complication is the Schneiderian membrane perforation (29). Regarding the volume, there are no studies determining the total volume obtained with this type of graft.

5.- Tuberosity: a very heterogeneous region among patients, with many anatomic factors affecting its size. For this reason, many studies do not describe its volume. It is a porous and medullary bone with great postoperative resorption (38,40). For this reason, it is not recommended to use it as a block, although many studies used it in that form (37,40). Postoperative resorption has not been observed in alveolar preservation. It is not recommended to use it in a particulate form (40). Its main advantage is the low complication rate (9,36), the complication reported being postoperative hematoma. Most studies call to use this region for small defects (38-40).

6.- Incisive fossa: there are few studies using the incisive fossa as a donor site. Nevertheless, it is a good donor site with few complications (31,32) and with the ability to regenerate small defects, used in particulate or block form. Regarding the volume, there are no studies determining the total volume obtained with this graft.

7.- Anterior nasal spine: donor site with a very scarce bone volume. It is difficult to extract the graft from this site and the main complication is the perforation of the Schneiderian membrane (33,34) Cho *et al.* (33) did not find aesthetic alterations related to the use of this type of graft. Nevertheless, this should be a first choice donor site to regenerate bone defects.

8.- Palate: donor site with abundant bone volume. It has been scarcely studied. The site where the graft should be extracted has not been concretely established, but both studies extracted the graft from the palatine of the site to be regenerated. A cadaver study Hassani and Khojasteh (47) considered as palatine donor the site between the maxillary central incisor and the maxillary second premolar. The medial limit was the incisive papilla and the basement membrane upper limit. These authors obtained an average volume of 2.02, a proper volume to regenerate medium defects. The main complications are basement membrane perforation (47) and possible damage to tooth structures (35,36), both avoidable if a complete radiologic study is conducted. We recommend further clinical trial to evaluate the performance of the graft over time because this site is easily accessible and has high bone volume and minimal complications.

9.- Mandibular torus: different studies show positive results when using a mandibular torus as a donor site, either in particulate or in block form. The great advantage is that the torus is an easily accessible and resection site. The studies analyzed do not describe complications in the donor site. The main disadvantage of this site is the limited quantity of bone available for use, but when available it is a very good alternative site to take into consideration.

Although many alternative donor sites have been studied, most sites grant scarce bone volume. They should be used as an alternative in small defects. In medium defects the only alternative sites to be used would be the zygomatic body, palatine site, and in some cases the anterior wall of the maxilla. But even so, it is difficult to see these sites as substitutes for the traditional ones because of the lack of studies supporting those techniques. It is very important to increase the clinical trials on alternative donor sites with higher quantity of bone. Nevertheless, nowadays surgical techniques as guided bone regeneration with absorbable or non-absorbable meshes (48,49), box technique (25), or sandwich osteotomy (50) are being used when the bone volume of the autologous bone to regenerate small defect is needed because of the mixture with some kind of bone substitute. For those techniques, it is a viable alternative to the aforementioned donor sites, where the prevalence of intraoperative and postoperative complications is low, with lower morbidity for the patient.

## Conclusions

Alternative donor sites to the chin and ramus are generally accessible sites with low morbidity, but they have low bone quantities. Because of this, their use is limited. Nevertheless, different regenerative techniques are currently being developed. They reduce the quantity of bone needed to regenerate bigger size defects; that, precisely, is their utility. However, a larger quantity of clinical trials supporting the clinical use and performance of these new sites are needed. Furthermore, we recommend conducting cadaver and radiologic studies to compare cortical and medullary surface areas, volumes and thicknesses between the sites to be used as grafts.
